# Symptomatic Cushing’s syndrome and hyperandrogenemia in a steroid cell ovarian neoplasm: a case report

**DOI:** 10.1186/s13256-016-1061-x

**Published:** 2016-10-12

**Authors:** Ramy Sedhom, Sophia Hu, Anupam Ohri, Dorian Infantino, Sara Lubitz

**Affiliations:** 1 Department of Internal Medicine, Rutgers Robert Wood Johnson Medical School, New Brunswick, NJ USA; 2 Division of Endocrinology, Metabolism & Nutrition, Department of Medicine, Rutgers Robert Wood Johnson Medical School, New Brunswick, NJ USA; 3 Department of Pathology and Laboratory Medicine, Rutgers Robert Wood Johnson Medical School, New Brunswick, NJ USA

**Keywords:** Cushing’s syndrome, Hyperandrogenemia, Steroid cell ovarian neoplasm, Ectopic cortisol

## Abstract

**Background:**

Malignant steroid cell tumors of the ovary are rare and frequently associated with hormonal abnormalities. There are no guidelines on how to treat rapidly progressive Cushing’s syndrome, a medical emergency.

**Case presentation:**

A 67-year-old white woman presented to our hospital with rapidly developing signs and symptoms of Cushing’s syndrome secondary to a steroid-secreting tumor. Her physical and biochemical manifestations of Cushing’s syndrome progressed, and she was not amenable to undergoing conventional chemotherapy secondary to the debilitating effects of high cortisol. Her rapidly progressive Cushing’s syndrome ultimately led to her death, despite aggressive medical management with spironolactone, ketoconazole, mitotane, and mifepristone.

**Conclusions:**

We report an unusual and rare case of Cushing’s syndrome secondary to a malignant steroid cell tumor of the ovary. The case is highlighted to discuss the complications of rapidly progressive Cushing’s syndrome, an underreported and often unrecognized endocrine emergency, and the best available evidence for treatment.

## Background

Ectopic Cushing’s syndrome is rarely seen with ovarian tumors. Steroid cell tumors are rare stromal tumors of the ovary first defined by Scully in 1979 [1]. They account for <0.1 % of ovarian tumors [2]. These tumors are associated with androgenic changes in 56–77 % of cases and Cushing’s syndrome in 6–10 % [3]. Owing to the rarity of these tumors, little is known regarding best treatment. In addition, the debilitating effects of hypercortisolism are often overlooked and undertreated. We report a case of a patient with a malignant steroid-secreting tumor causing rapid onset of virilization and Cushing’s syndrome, and she was not amenable to aggressive medical therapy.

## Case presentation

A 67-year-old nulliparous white woman with no prior medical history or pertinent family history presented to our hospital with a 4-month history of hirsutism, deepening voice, weight gain, easy bruising, hair thinning, and chest redness. Her physical examination revealed abdominal swelling, pedal edema, and excessive hair growth. She had new-onset hypertension, with an elevated blood pressure of 153/78 mmHg. Initial laboratory test results revealed a serum glucose level of 543 mg/dl (normal 70–100 mg/dl), a potassium level of 2.5 mg/dl (normal 3.5–5 mg/dl), and a bicarbonate level of 44.8 mEq/L (normal 24–32 mEq/L). Her hemoglobin A1c was 9.2 %, compared with 5.4 % when checked only 6 months prior. Further hormonal evaluation revealed a testosterone concentration >800 ng/dl (normal 8–60 ng/dl), a dehydroepiandrosterone level of 243 ng/ml (normal <145 ng/ml), a luteinizing hormone concentration <0.2 IU/L (normal 15.9–54 IU/L), and a follicle-stimulating hormone level <0.7 IU/L (normal 16.7–136.4 IU/L). The patient’s 24-h urine cortisol was 273 μg/24 h (normal <45 μg/24 h). Dexamethasone 1 mg failed to suppress her morning cortisol, which was 33 mg/dl (normal <5 mg/dl). Her aldosterone concentration was <4 ng/dl (normal 0–21 ng/dl), and her renin level was 1.2 ng/ml (normal 0.6–3.0 ng/ml). Magnetic resonance imaging (MRI) of the her abdomen and pelvis revealed a 9.4 × 5.8 × 7.9-cm ovarian mass with ascites and diffuse abdominal metastasis. Ovarian hormone testing disclosed a CA-125 level of 742.6 U/ml (normal 0–35 U/ml), an inhibin A level of 11 pg/ml (normal <2.1 pg/ml), and an inhibin B level of 5060 pg/ml (normal <10 pg/ml). She was started on lisinopril 10 mg, furosemide 20 mg, hydrochlorothiazide 12.5 mg, and potassium chloride 10 mEq daily, as well as metoprolol 25 mg twice daily.

An exploratory laparotomy of the right ovary revealed a lobulated mass measuring 9.8 cm in its greatest dimension. The tumor extended across to the left ovary and involved the bladder, periovarian tissue, anterior abdominal wall, and cecum. Liver metastasis was also noted. Because of the extent of the patient’s disease, a complete resection was not attempted. A hysterectomy, bilateral salpingo-oophorectomy, and tumor debulking were performed.

The cut surface of the tumor was orange with hemorrhage. Histologically, polygonal cells and abundant cytoplasm were seen, ranging from eosinophilic to granular, with brisk mitotic activity (22 mitoses per 10 high-power fields [HPF]). The tumor demonstrated a solid and trabecular pattern of growth and focal myxoid stroma. Reinke crystals were absent. The tumor stained positive for inhibin, calretinin, and MART-1 and negative for chromogranin, S-100, WT-1, CK7, and CD99. Based on these findings, a diagnosis of ovarian steroid cell tumor was made.

Postoperatively, the patient’s testosterone (90 ng/dl) and urinary cortisol (656 μg/24 h) were elevated with adrenocorticotropic hormone (ACTH) 12 pg/ml. No prior ACTH measurement was available. Spironolactone and ketoconazole were started in response to hypokalemia, edema, and high cortisol. Spironolactone was titrated up to 400 mg daily and ketoconazole to 1200 mg daily with persistent hypokalemia. Mitotane 1500 mg daily and mifepristone 300 mg daily were added. Disease burden continued to progress as urine cortisol remained elevated at 1056 μg/24 h, with refractory hypokalemia.

Complications postoperatively included wound dehiscence with methicillin-resistant *Staphylococcus aureus* infection requiring surgical debridement and intensive care unit admission for pneumonia. She developed delirium, depression, and malnutrition. Her poor functional status prohibited conventional chemotherapy. A decision was made to provide comfort care, and she subsequently died in the hospice (Table 1).

**Table 1:**
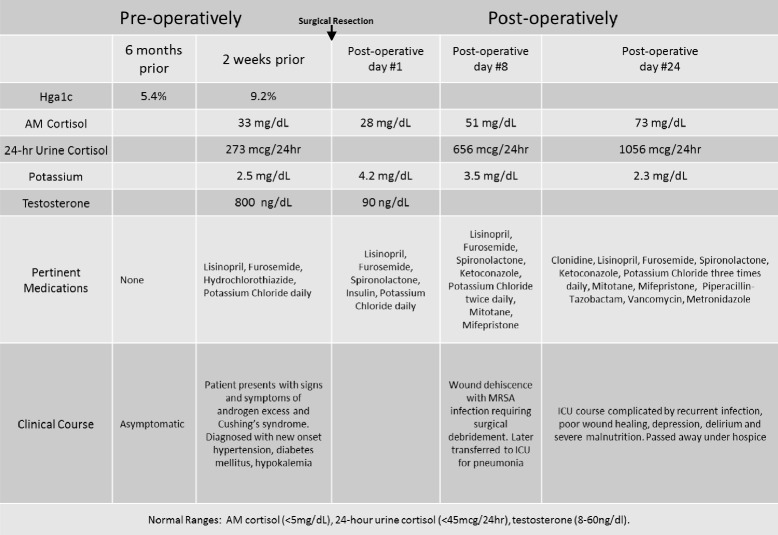
The patient’s biochemical, hormonal, and clinical profiles pre- and postoperatively

## Discussion

In 1912, Harvey Cushing described a case of a young, obese woman with thin extremities who had hirsutism, amenorrhea, and diabetes; her disease would come to bear Cushing’s name [4]. The most discriminating features of Cushing’s syndrome are easy bruising, facial plethora, proximal myopathy, and purple striae. Nonsuppressible hypercortisolism confirms the diagnosis.

Our patient had historical and objective features suggestive of Cushing’s syndrome. However, many clinical signs and biochemical features were also suggestive of hyperandrogenism. Consideration of an androgen-secreting neoplasm was based on the patient’s plasma androgen level. Testosterone levels >150 ng/dl or dehydroepiandrosterone sulfate levels >700 μg/dl suggest an androgen-secreting neoplasm [5].

Cushing’s syndrome is rarely associated with ovarian neoplasms. Though the incidence of Cushing’s syndrome due to ovarian neoplasms is unknown, authors of case reports have described mechanisms of ectopic ACTH secretion, ectopic production of ACTH-like peptides, corticotropin-releasing factor, and cortisol [6, 7].

Ovarian steroid cell tumors are classified into three subtypes: stromal luteoma, Leydig cell tumor, and steroid cell tumor not otherwise specified (NOS). Steroid cell tumor NOS is the most common of the three subtypes, accounting for 60 % of cases [2, 8]. Steroid cell tumors NOS have an undefined lineage and cannot be categorized as either stromal luteomas or Leydig cell tumors. The mean age at diagnosis is 43 years, though patients have ranged from 3 to 93 years of age [9]. A case series of 63 patients reported by Hayes and Scully documented that 94 % were unilateral and a majority (75 %) were capable of sex steroid hormone production. Evidence of androgen excess was seen in 56 % of patients, estrogen excess in 6 %, and cortisol production in 6–10 %. In adults, approximately one-fourth of steroid cell tumors were malignant. In their original paper, Hayes and Scully did not describe any patients as having both excess androgen and cortisol, making the case of our patient of particular interest [2].

The clinical manifestations of steroid cell tumor NOS are similar to both stromal luteomas and Leydig cell tumors and are associated with hormonal activity and virilization. Common virilizing findings include hirsutism, acne, deep voice, and alopecia. Estrogenic effects are not uncommon and include menorrhagia, postmenopausal bleeding, and endometrial changes. Symptoms of Cushing’s syndrome include abdominal pain, distention, and bloating, but they are seen in only 6–10 % of cases [1, 2, 9, 10].

The majority of steroid cell tumors have either benign or low-grade behavior [11, 12]. Most are diagnosed at a younger age in an early stage, are small, and are hormonally inactive. Nulliparity has not been identified as an epidemiological risk factor for steroid cell tumors. Typically, these tumors do not recur or metastasize. Therefore, surgical resection is considered definitive treatment. Of the steroid cell tumor cases found to be clinically malignant, only 20 % showed metastatic lesions outside the ovary at the time of surgery. When metastasis is present, lesions are typically localized to the peritoneal cavity. Metastasis is rarely found at distant sites [2, 9–11]. The liver lesion noted in our patient is the first in the literature, to our knowledge.

Malignancy in steroid cell tumors is strongly suggested by the following pathological features: two or more mitotic figures per 10 HPF, necrosis, a diameter >7 cm, hemorrhage, and grade 2 or 3 nuclear atypia [2]. Our patient’s case satisfied multiple criteria for malignancy, including necrosis, hemorrhage, atypia, mitotic features, and a tumor diameter >7 cm.

**Fig. 1:**
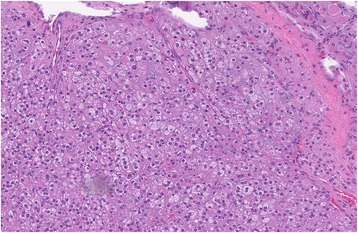
Hematoxylin and eosin stain with characteristic microscopic appearance and histology of steroid cell tumor not otherwise specified. Seen are large aggregates of polygonal to round tumor cells having distinct cell borders, central nuclei, and prominent nucleoli. Also present are delicate fibrous bands. Reinke crystals are absent

**Fig. 2:**
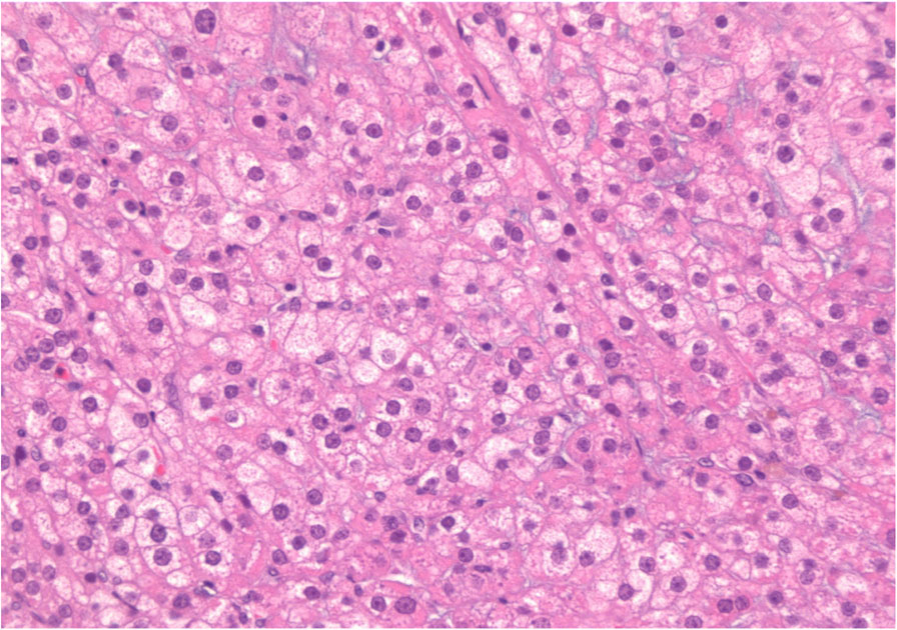
Higher-power view of Fig. 1. The cytoplasm of the tumor cells shows variably sized clear vacuoles, representing fat material

The diagnosis is made histologically as first defined by Scully [2]. Steroid cell tumors NOS have two types of polygonal cells that differ by cytoplasmic appearance: eosinophilic or vacuolated. Absence of Reinke crystals differentiates them from Leydig tumors. The prior nomenclature of “lipid” cell tumors was misleading because many tumors had little lipid present [3]. The average diameter for malignancy is 8.5 cm, and the cut surface is yellow-orange. Microscopically, cells are polygonal, with central nuclei and prominent nucleoli [11]. Cells are positive for fat stains [9, 10, 12]. Our patient had gross and microscopic findings consistent with ovarian steroid cell tumor NOS (Figs. 1 and 2).

**Fig. 3:**
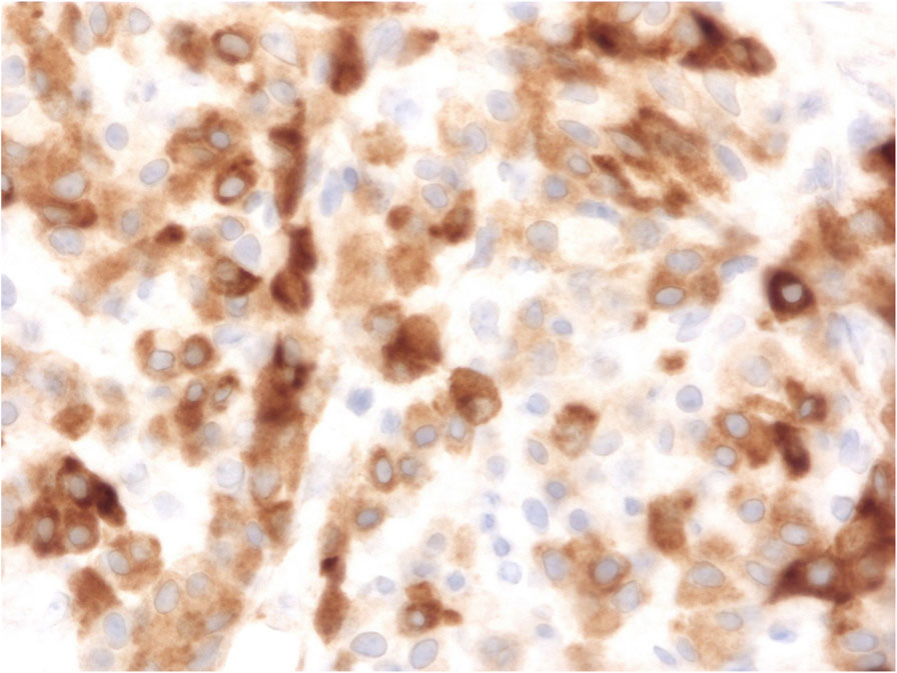
Immunohistochemical staining revealing inhibin positivity in the tumor cells, classifying the tumor as a sex cord-stromal tumor

**Fig. 4:**
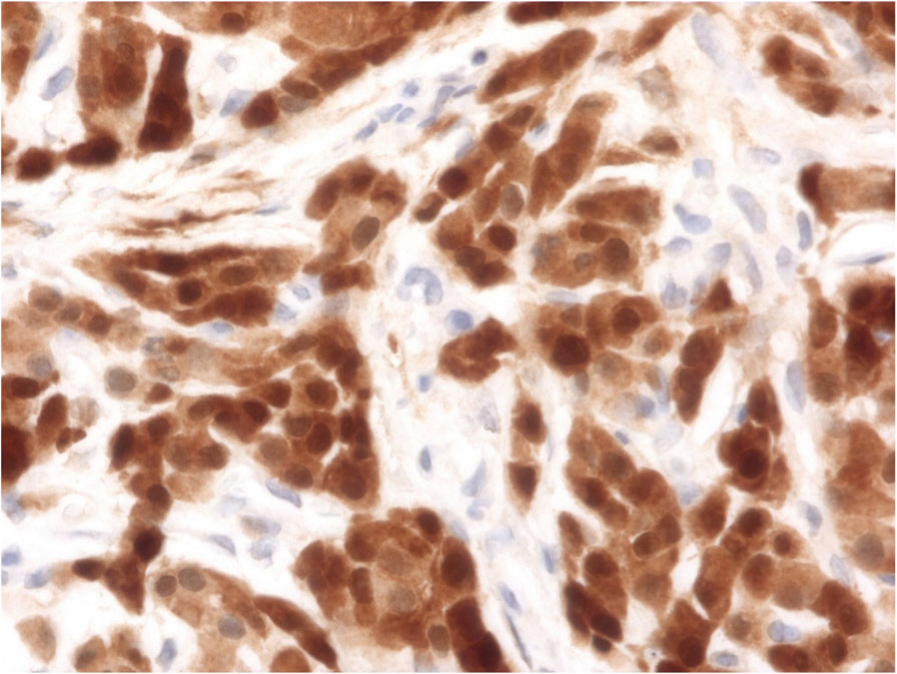
Immunohistochemical staining revealing calretinin positivity in the tumor cells, revealing its steroid cell-secretory nature

Immunohistochemistry aids in diagnosis, with inhibin and calretinin differentiating sex cord-stromal from non-sex-cord tumors. Seventy-five percent of cases are vimentin-positive [10, 13]. Inhibin positivity classifies the tumor as a sex cord-stromal tumor (Fig. 3). Positive calretinin defines its steroid cell-secretory nature (Fig. 4). Negative immunostains rule out other malignancies. In our patient, negativity of WT-1 ruled out granulosa cell tumors, and negative CK7 and CK99 ruled out a neuroectodermal origin [9–11, 13].

On imaging studies, steroid cell tumors are typically unilateral and solid. Cystic changes or necrosis is possible. Most are small and thus frequently undetected by ultrasound or computed tomography [14]. MRI is the best modality for tumor identification, with high signal intensity on T1-weighted images due to lipid content and intense enhancement with gadolinium reflecting high vascularity of the tumors [14].

The best treatment for patients diagnosed with steroid-secreting ovarian sex cell tumors has not been described. Most of these tumors are diagnosed at an early stage and do not recur or metastasize; thus, little is known about response to therapy, and there is no recommended standard of care.

Treatment of advanced ovarian steroid cell tumors is primarily surgical. Following cytoreductive surgery, adjuvant chemotherapy is used, though no formal recommendations have been made [10, 12, 15]. The response is typically short-lived. Our patient was not a candidate for chemotherapy. Her functional status was limited by her tumor burden and, more importantly, the hormonal manifestations of uncontrolled Cushing’s syndrome.

Rapid, aggressive Cushing’s syndrome is, and should be considered as, an “endocrine emergency.” It is a rare presentation of ovarian tumors and is highlighted in the case of our patient. Inadequately treated Cushing’s syndrome is associated with a three- to fivefold higher mortality [16]. Prompt, aggressive treatment, with multiple agents directed at both decreasing cortisol production and dampening its effects on end targets, is mandated. There are numerous agents available to meet these treatment goals. Their mechanism of action and duration of onset are important to consider and are discussed next.

Ketoconazole is an imidazole derivative originally developed as an antifungal agent. It blocks sex steroid and cortisol synthesis by multiple mechanisms, including inhibiting 11β-hydroxylase, 17α-hydroxylase, and C17,20 lyase enzymes. It can also inhibit ACTH secretion by corticotroph tumor cells [15, 17, 18]. Ketoconazole decreases cortisol in 80 % of patients. Urinary free cortisol is normalized in up to 49 % of patients [18]. However, ketoconazole takes weeks to decrease cortisol; thus, it has limited use as a single agent in the treatment of rapidly progressive Cushing’s syndrome. The treatment of Cushing’s syndrome is an off-label use for ketoconazole.

Metyrapone is an 11β-hydroxylase inhibitor that blocks the final step in cortisol synthesis: conversion of 11-deoxycortisol to cortisol [17]. It is potent and short-acting. Cortisol levels are decreased in 75 % of patients. Urinary free cortisol is improved as early as 1 week following treatment. Data regarding the long-term efficacy of metyrapone remains scarce [17]. Therefore, metyrapone is used as adjunctive therapy. Its use for treatment of Cushing’s syndrome is an off-label use. Additionally, increased 11-deoxycortisol levels cross-react with serum and urine immunoassays, resulting in elevated cortisol levels, making accurate assessment of disease difficult [17, 18].

Mitotane is a derivative of dichlorodiphenyltrichloroethane and reduces cortisol production by blocking cholesterol side-chain cleavage and 11β-hydroxylase [15, 17, 18]. It stimulates CYP3A4 expression, reducing cortisol bioavailability. Its full effect is not seen for 3 months. Mitotane has demonstrated efficacy in adrenal adenocarcinoma and is used to treat all forms of hypercortisolism, including unresectable Cushing’s disease. While 80 % of patients achieve normalization of urinary markers, 60 % relapse after therapy withdrawal [18]. Mitotane also causes changes in hormone-binding globulin, causing total hormone measurements to be inaccurate. As a result, free urinary and serum cortisol is the best index of response [17, 18].

Mifepristone (RU-486) is the only U.S. Food and Drug Administration-approved medication for Cushing’s syndrome of any cause. It is a potent glucocorticoid and progesterone receptor antagonist, blocking cortisol at the tissue level. The block in glucocorticoid action leads to negative feedback at the hypothalamic-pituitary level, resulting in a rise in ACTH and cortisol [17]. Recently published data show clinical improvement in 87 % of patients, with improvements in blood sugar, diastolic blood pressure, and weight loss [19]. It is also used in severe hypercortisolism when the chance of surgical cure is low [17, 19]. Currently, there is no biochemical marker to monitor drug effectiveness or assist in dose titration [15, 17].

Etomidate is an imidazole derivative used for anesthesia induction. It reduces cortisol production by blocking cholesterol side-chain cleavage, aldosterone synthase, and 11β-hydroxylase. Case reports show successful use of etomidate as a short-term treatment in critically ill patients with Cushing’s syndrome. It has a rapid onset of action, with cortisol levels decreasing within 12 h [20]. As a continuous intravenous infusion, it is an ideal option for rapid control of hypercortisolemia. However, its use is limited to the intensive care unit, where serum cortisol, potassium, and level of sedation are closely monitored [20, 21]. More studies are needed to better elucidate the pharmacokinetics and adverse effects of etomidate to create treatment protocols. The treatment of Cushing’s syndrome is an off-label use of etomidate.

## Conclusions

The case of our patient highlights a rare coexistence of Cushing’s syndrome and hyperandrogenemia due to a malignant steroid cell neoplasm of the ovary. Rapidly progressive Cushing’s syndrome presented a unique therapeutic challenge, with the biochemical effects of hypercortisolism leading to rapid morbidity and mortality. When surgery fails to reverse hypercortisolism, medical treatment can suppress cortisol overproduction and its end-target effects, improving clinical status.

In retrospect, a more aggressive approach using multiple potent, short-acting agents may have improved the patient’s outcome. Careful monitoring and treatment by clinicians familiar with medications’ mechanisms of action are essential. The case of our patient underscores the need for further research into the biology of this tumor and a targeted approach for treating severe hypercortisolism. This addition to the literature highlights a rare malignancy and an unrecognized endocrine emergency.
